# Supplementary material for: Shared Treg dysregulation underlies psoriasis vulgaris and atopic dermatitis through the PHF19-PRC2 epigenetic axis
Source: Front Immunol. 2026 Aug 20;17:1932788. doi: 10.3389/fimmu.2026.1932788 (PMC13538097; doi:10.3389/fimmu.2026.1932788)
Supplement: Supplementary file 3 [file DataSheet3.zip › Additional files/Additional file 7/66.FAM200B.funnel.pdf]

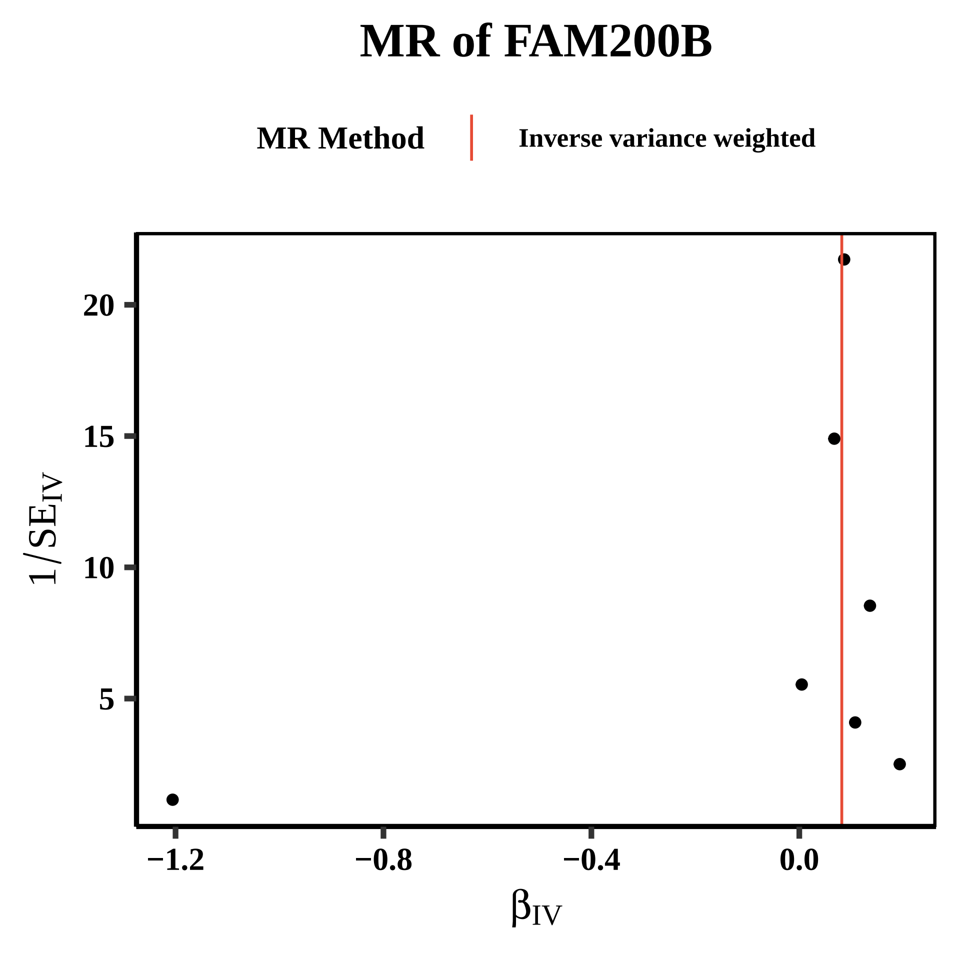
MR of FAM200B
MR Method
Inverse variance weighted
20
15
10
5
1/SE_IV
−1.2
−0.8
−0.4
0.0
β_IV
